# Comparison of surgical field visibility during propofol or desflurane anesthesia for middle ear microsurgery

**DOI:** 10.1186/s12871-019-0759-x

**Published:** 2019-05-24

**Authors:** Xia Yuan, Tingjie Liu, Chunbo Hu, Xia Shen

**Affiliations:** Department of Anesthesiology, The Eye, Ear, Nose and Throat Hospital, Fudan University, 83 fenyang road, Shanghai, 200031 China

**Keywords:** Anesthetics, inhalation, Desflurane, Anesthetics, intravenous, Propofol, Hypotension, controlled, Surgery, Otolaryngology

## Abstract

**Background:**

To compare surgical field visibility between patients given propofol/remifentanil (PR) or desflurane/remifentanil (DR) anesthesia.

**Methods:**

A total of 80 adult patients undergoing middle ear microsurgery due to cholesteatoma otitis media with American Society of Anesthesiologists physical status I and II were randomly assigned to the PR or DR groups. The depth of anesthesia was titrated to maintain a Bispectral index (BIS) between 40 and 50. Remifentanil was titrated to maintain the mean blood pressure within ±30% change of the pre-induction value. Surgical field visibility was rated at several timepoints by the surgeons using the Boezaart scores.

**Results:**

Average Boezaart scores for surgical field visibility at different time points were < 2 in both PR and DR groups. Surgical field visibility score was lower in the PR group than in the DR group. Requirement for remifentanil was higher in the PR group (850 (488/1330) μg) than in the DR group (258 (143/399) μg, *P* < 0.0001). The site effect concentration of remifentanil was higher in the PR group (3.6(2.8/5.0)ng/ml) than in the DR group (1.7 (1.0/1.6) ng/ml, *P* < 0.0001). Hemodynamic profile (i.e., heart rate and mean blood pressure) was similar between groups (*P* > 0.05). Extubation time (PR group, 21 min vs. DR group, 19 min; *P* = 0.199) and post-anesthesia care unit time (PR group, 37 min vs. DR group, 34 min; *P* = 0.324) were comparable between groups.

**Conclusion:**

Although PR anesthesia resulted in lower surgical field visibility scores than DR anesthesia, both groups had scores < 2, meaning no clinical differences between the two groups. DR provided acceptable operative conditions as well, albeit more remifentanil consumption was noted in the DR group.

**Trial registration:**

China Clinical Research Information Service, ChiCTR-1,800,015,537. Registered 5 April 2018. Date of enrolment of the first participant to the trial: 2 May 2018.

## Background

Optimizing the visualization of anatomy during middle ear microsurgery can decrease the duration of surgery, reduce blood loss, and improve surgical outcomes [1]. Therefore, several procedures including head-up tilt maneuver, locally applied vasoconstriction, controlled hypotension [2], pharmacological agents [3], and manipulation of general anesthesia [4] have been employed to improve the visual quality of the surgical field.

Anesthetic agents can influence surgical field visibility and the amount of blood loss through pharmacological effects on vasodilation and heart rate. There is consensus that anesthesia with propofol provides better surgical field visibility and less blood loss than anesthesia with isoflurane or desflurane [5, 6]. Consistent with findings that surgical conditions are correlated with intraoperative heart rate rather than mean blood pressure [6–8], propofol anesthesia is associated with a lower intraoperative heart rate and has been found to result in better surgical field visibility than inhalation anesthesia [6].

Desflurane possesses a very low blood-gas partition coefficient (0.45), allowing fast awakening and recovery [9]. It is commonly used during clinical practice to ensure efficient operating room management. However, desflurane tends to stimulate circulation (i.e., increase heart rate) and thus may not be appropriate for middle ear surgery, which demands optimal surgical field visibility. However, in our otology surgical center, desflurane is widely used with little complaint from surgeons regarding impaired surgical field visibility. To our knowledge, few studies have compared middle ear surgical field visibility between propofol/remifentanil (PR) and desflurane/remifentanil (DR) anesthesia. We hypothesized that propofol/remifentanil anesthesia may provide better surgical condition than desflurane/remifentanil anesthesia during middle ear microsurgery. Therefore, we conducted a double-blind, randomized controlled study to assess the effects of desflurane on surgical field visibility, intraoperative hemodynamic profile, and patient recovery from middle ear microsurgery.

## Materials and methods

This study was registered in the Chinese Clinical Trial Registry (ChiCTR1800015537). The Institutional Review Board (Shanghai Eye, Ear, Nose, and Throat Hospital affiliated with Fudan University) approved the study procedures, and each enrolled patient provided written informed consent.

Adult patients with American Society of Anesthesiologists physical status I and II scheduled for middle ear surgery due to cholesteatoma otitis media were consecutively recruited. Patients were randomly assigned to the propofol/remifentanil (PR) or desflurane/remifentanil (DR) group by computer-generated allocation. Patients were excluded if they were receiving cardiovascularly active drugs or drugs related to coagulation (e.g., warferin, heparin, enoxiparin, NSAID or aspirin).

Upon arriving to the operating room, non-invasive monitoring of arterial blood pressure, pulse oximetry, electrocardiography, and bispectral index (BIS) monitoring (BIS VISTA Monitoring System; Aspect Medical Systems, Inc., Norwood, MA, USA) was established. Anesthesia was induced by intravenous 2 mg/kg propofol (Fresenius Kabi, Beijing, China), 0.3 μg/kg remifentanil (Yichang Renfu Pharmaceutical, Yichang, China), and 0.6 mg/kg rocuronium (Hameln Pharmaceuticals GmbH, Hameln, Germany). After flexible laryngeal mask insertion, anesthesia was maintained with propofol/remifentanil (PR) or desflurane/remifentanil (DR). Patients in the PR group received an effect site target-controlled infusion of propofol based on Schnider’s pharmacokinetic model [10] and remifentanil based on Minto’s model [11], delivered using a commercial pump (Orchestraw Base Primea, Fresenius Vial, Brezins, France). Effect site concentration was 2–6 μg/ml for propofol. For patients in the DR group, anesthesia was maintained with 4–8% desflurane. The effect site concentration of remifentanil was 1–8 ng/ml for both groups. Mean blood pressure was maintained within ±30% change of the pre-induction value. Ephedrine was needed for any decrease in mean blood pressure lower than 30% from the patient’s pre-induction value.

Patients received mechanical ventilation in pressure-controlled mode with a tidal volume of 8 ml/kg at a rate of 10 breaths per min to provide an end-tidal CO_2_ concentration of 35–45 mmHg. The carrier gas flow for both groups consisted of oxygen and air (FiO_2_ 0.5) during anesthesia maintenance. Intravenous 1 mg/kg parecoxib (Pfizer Pharmaceuticals, Beijing, China), and 0.01 mg/kg hydromorphone hydrochloride (Yichang Renfu Pharmaceutical) were given at the end of surgery for postoperative analgesia, and 0.15 mg/kg ondansetron hydrochloride and 0.1 mg/kg dexamethasone were given to prevent postoperative nausea and vomiting.

Surgery was performed by one of three surgeons with subspecialty training in otology using a similar stepwise technique. The surgeons were blinded to the anesthesia type by shielding the vaporizer and propofol and remifentanil syrings. Patients’ heads were positioned 15° higher than their bodies for the entire procedure. Attending surgeons who were blind to treatment group rated surgical field visibility from 0 to 5 according to the Boezaart grading scale where 0 denotes the best and 5 the worst visibility [12]. Surgical field visibility was recorded at four time points: skin incision, bone drilling, clearance of cholesteatoma, and laying of fascia. Hemodynamic profile was recorded at the following time points: arrival to the operating room, 10 min after skin incision, completion of drilling, completion of clearance of cholesteatoma, completion of laying of fascia, extubation, 10 min after extubation, and post-anesthesia care unit (PACU) discharge.

Anesthesia time was defined as the time from anesthesia induction to anesthetic discontinuation. After surgery, patients were transferred to the PACU for monitoring and management by an attending anesthesiologist who was blinded to the group assignment. This anesthesiologist was blinded to the study, as was the research assistant who recorded extubation time (time from the end of surgery to extubation), PACU time (time between extubation and achieving a modified Aldrete score [13] > 9), postoperative pain (visual analogue scale (VAS) from 0 to 10 with 0 = no pain and 10 = worst pain imaginable), and postoperative nausea and vomiting (PONV) episodes. If VAS score was > 4, a rescue analgesic (intravenous 0.01 mg/kg hydromorphone hydrochloride) was given. Intravenous (10 mg) metoclopramide was given when needed as an antiemetic.

The primary outcome was surgical field visibility. Secondary outcomes were requirement for remifentanil, hemodynamic profile, and recovery time. Based on a pilot study of 10 adult patients receiving DR anesthesia, mean surgical field visibility was 2 (standard deviation, 1) during clearance of the focal lesion during middle ear microsurgery. Based on an estimate of 40% improvement in surgical field visibility, we calculated that 40 patients in each group were needed to provide statistical power of 80% at a 5% significance level. Remifentanil usage is expressed as the median (first/third quartiles) and was analyzed using a Mann-Whitney rank sum test. Other continuous variables are expressed as mean ± standard deviation (SD) and were analyzed using Student’s *t*-tests. Categorical data were analyzed using χ^2^ tests or Fisher’s exact tests. Repeated measures ANOVA was used to analyze changes in hemodynamic profiles (heart rate and mean blood pressure) over time. Statistical significance was set at *P* < 0.05.

## Results

**Fig. 1:**
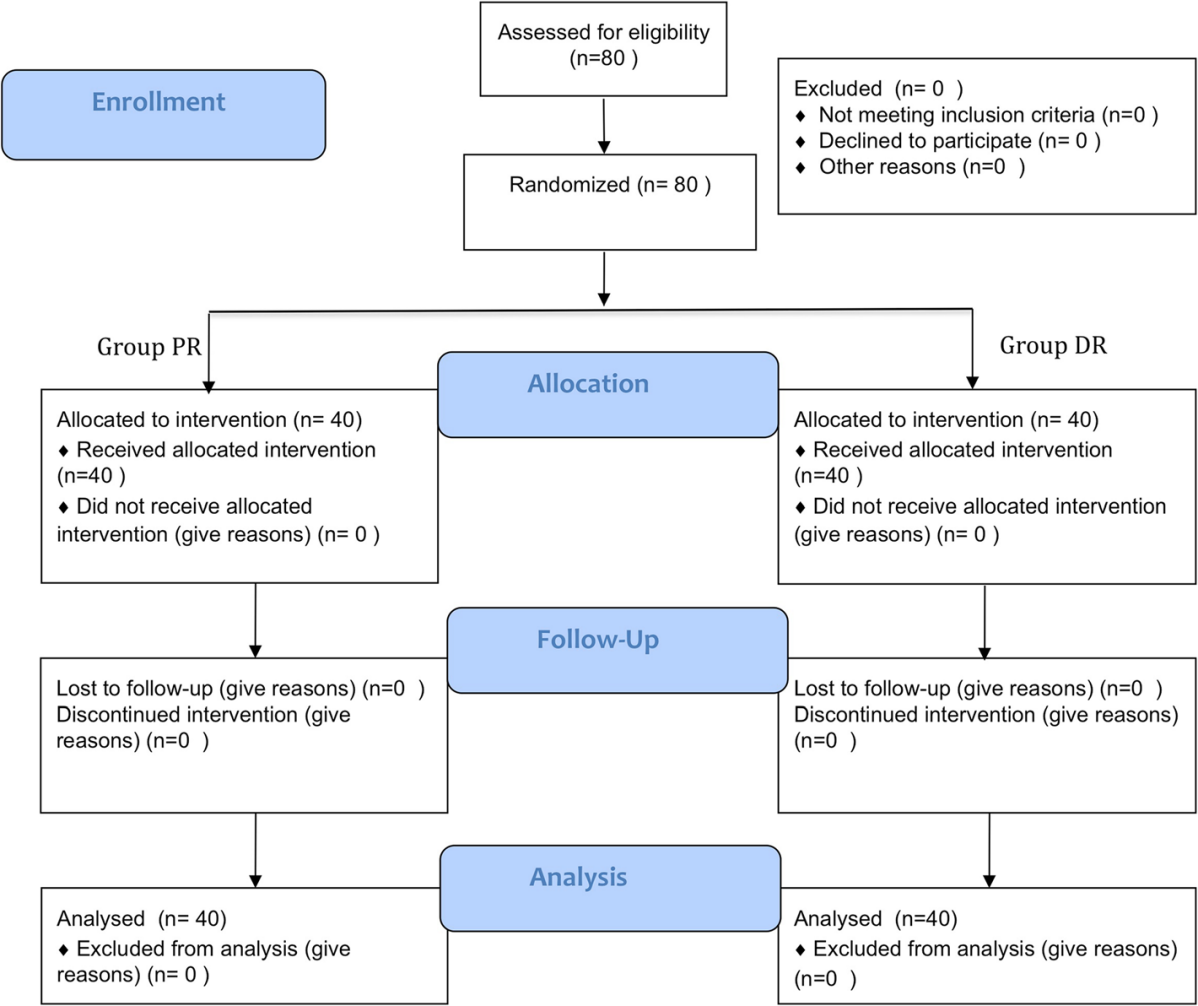
CONSORT flow diagram. PR, propofol/remifentanil; DR, deflurane/remifentanil

Eighty patients were enrolled in and completed the study (Fig. 1). Gender, age, height, weight, body mass index, anesthesia and surgery times, and distribution of the three surgeons were similar between PR and DR groups (Table 1).

**Table 1:** Demographic characteristics of patients

	Group PR (*n* = 40)	Group DR (*n* = 40)
Sex (M/F)	21/19	19/21
Age (yr)	43.1 (11.3)	45.7 (11.4)
Weight (kg)	66.4 (11.8)	63.9 (10.2)
Height (cm)	165.2 (7.1)	166.2 (8.0)
Body mass index (kg•m^− 2^)	24.2 (3.5)	23.1 (2.8)
ASA classification (I/II)	30/10	32/8
Operator (1/2/3)	16/13/11	14/16/10
Surgery duration (min)	89.1 (34.4)	83.3 (33.0)
Anesthesia duration (min)	103.2 (36.1)	92.5 (31.0)
Baseline BIS value	97 (1)	97 (1)

Values are presented as mean ± standard deviation or number of patients. *PR* propofol/remifentanil, *DR* desflurane/remifentanil, *ASA* American Society of Anesthesiologists, *BIS* bispectral index

**Fig. 2:**
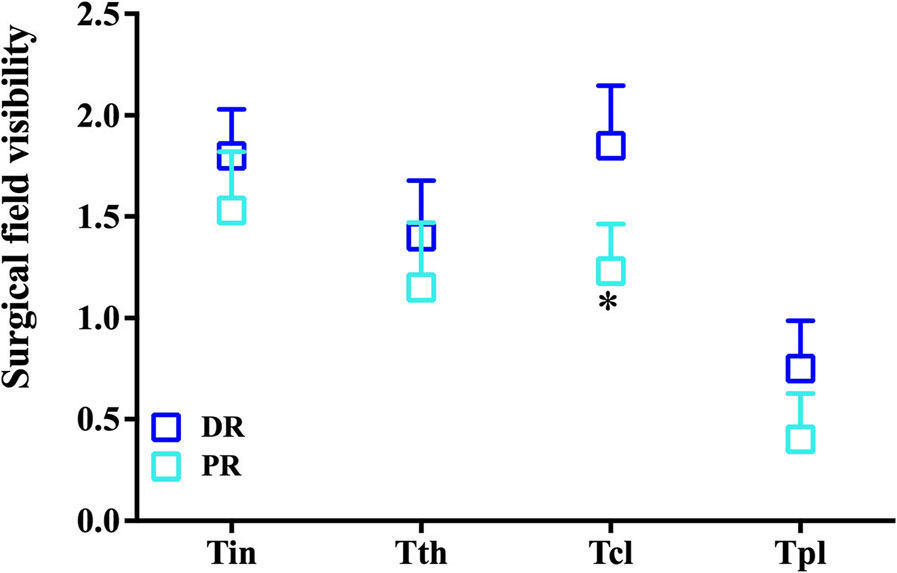
Surgical field visibility at different time points. Average Boezaart scores for surgical field visibility at different time points were < 2 in both propofol/remifentanil (PR) group and desflurane/remifentanil (DR) group. Surgical field visibility was significantly better in the PR group than in the DR group during clearance of cholesteatoma. T_ln,_ at skin incision; T_dr,_ during bone drilling; T_cl._ during clearance of cholesteatoma; T_pl,_ during laying of fascia. *P < 0.05

Average surgical field visibility score was < 2 for both groups (Fig. 2). The PR group had lower surgical field visibility scores (i.e., better visibility) than the DR group during clearance of the focal lesion (1.22 vs. 1.85, *P* = 0.001). The surgical field visibility scores were also lower at skin incision, bone drilling, and laying of fascia in PR group, but the differences were not significant (*P* > 0.05).

**Fig. 3:**
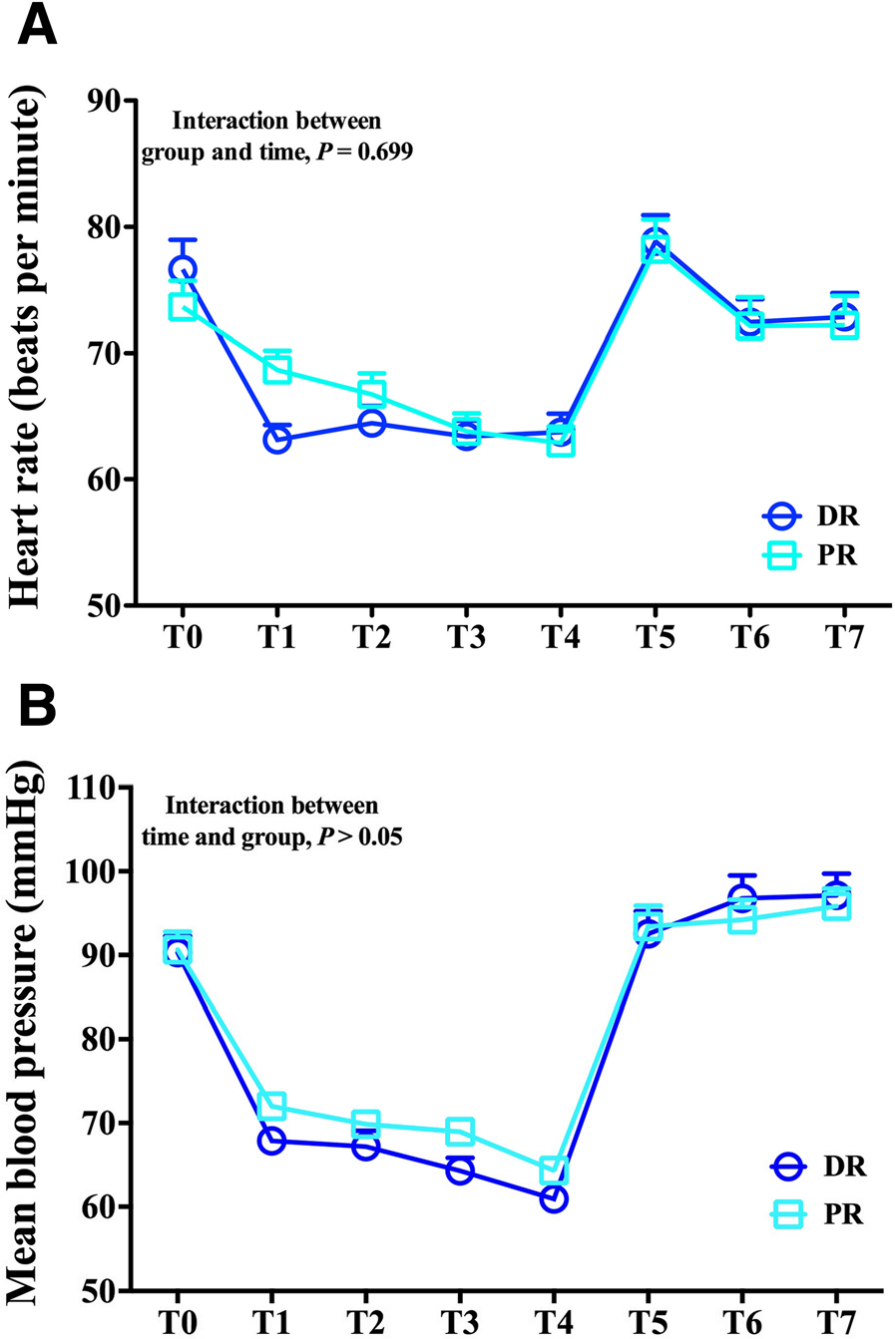
Hemodynamic profile. Changes in (**a**) heart rate and (**b**) mean blood pressure at different time points. Repeated measures ANOVA showed no differences in hemodynamics over time between the propofol/remifentanil (PR) and desflurane/remifentanil (DR) groups. T_0_, upon arrival in the operating room; T_1_, 10 min after surgical incision; T_2_, completion of drilling; T_3_, completion of clearance of cholesteatoma; T_4_, completion of laying fascia; T_5_, at extubation; T_6_, 10 min after extubation; T_7_, at PACU discharge

Regarding hemodynamic profile, mean blood pressure was maintained within ±30% change of pre-induction values. Patients in both groups showed significant decreases in mean blood pressure during surgery (T_1_–T_4_), but there were no differences between groups (*P* > 0.05; Fig. 3). After surgery (T_5_–T_7_), mean blood pressure returned to baseline values. Heart rate also decreased during surgery (T_1_–T_4_) and returned to baseline values after surgery (T_5_–T_7_), with no differences between groups (*P* > 0.05).

Intraoperative BIS was comparable between groups (*P* > 0.05; Table 2). Patients in the PR group required more remifentanil than patients in the DR group (PR group, 850 (488/1330) μg vs. DR group, 258 (143/399) μg, *P* < 0.0001). The site effect concentration of remifentanil was higher in the PR group (PR group, 3.6(2.8/5.0) ng/ml vs. DR group, 1.7 (1.0/1.6) ng/ml, *P* < 0.0001). The mean propofol site effect concentration was 3.4 μg/ml. The mean desflurane MAC was 0.8. Intraoperative ephedrine was required in a similar number of patients in each group (*P* > 0.05). Extubation time (PR group, 21 min vs. DR group, 19 min; *P* = 0.199) and PACU times (PR group, 37 min vs. DR group, 34 min; *P* = 0.324) were comparable between groups. One patient in each group required pain rescue in the PACU, respectively. No patient had PONV episodes in the PACU.

**Table 2:** Perioperative variables

	Group PR (*n* = 40)	Group DR (*n* = 40)	*P*-value
Intraoperative			
Remifentanil dose (μg)	850 (488/1330)	258 (143/399)	< 0.0001
Remifentanil effect-site concentration (ng/ml)	3.6 (2.8/5.0)	1.7 (1.0/1.6)	< 0.0001
Propofol effect-site concentration (μg/ml)	3.4(0.6)		
Desflurane MAC		0.8 (0.1)	
Intraoperative BIS value	44 (0.3)	43 (0.3)	0.515
Ephedrine usage	9	6	0.568
PACU			
Extubation time (min)	21 ± 11	19 ± 6	0.199
PACU time (min)	37 ± 14	34 ± 8	0.324
Analgesic rescue	1	1	1
PONV episodes	0	0	1

Values are reported as mean ± standard deviation, median (first/third quartiles), or number of patients; *PR* propofol/remifentanil, *DR* desflurane/remifentanil, *BIS* bispectral index, *MAC* minimal alveolar concentration, *PACU* post-anesthesia care unit, *PONV* postoperative nausea and vomiting

## Discussion

We found that the average surgical field visibility score was < 2 for both groups while the patients in propofol/remifentanil (PR) anesthesia group had lower surgical field visibility scores than those in desflurane/remifentanil (DR) anesthesia group during middle ear surgery. More remifentanil was needed in the PR group. The two anesthesia techniques were associated with similar hemodynamic profiles during surgery.

This confirms previous findings that propofol anesthesia allows better surgical field visibility and less blood loss than inhalation anesthesia [5, 6]. Some studies report that surgical conditions are correlated with intraoperative heart rate rather than mean blood pressure [6–8]. However, we found no differences between groups in heart rate or mean blood pressure during surgery. These findings suggest that when well controlled, heart rate itself is not the determination of operating conditions and that other mechanisms are in play.

Desflurane allows fast awakening and recovery due to its very low blood-gas partition coefficient (0.45) [9]. It is commonly used during clinical practice to ensure efficient operating room management. Although desflurane has been used for ear, nose, and throat surgery due to its beneficial effect on recovery [14], it tends to increase heart rate more than 1 minimal alveolar concentration (MAC) [9]. In our study, the mean MAC of desflurane to maintain an appropriate depth of anesthesia (i.e., mean BIS value of 43) was 0.8. We did not observe an increase in heart rate in patients receiving desflurane, perhaps due to remifentanil usage, as opioids can eliminate the stimulatory effect of desflurane on the circulation system [9].

We routinely use remifentanil during middle ear surgery in our clinic because of its analgesic effect and reduction of middle ear blood flow, consistent with Degoute et al. [15, 16]. Degoute et al. found that remifentanil/propofol anesthesia was effective in reducing middle ear blood flow and providing good surgical conditions for adult patients during tympanoplasty in a small sample size study [15]. Later they found that when combined with sevoflurane, an inhalation anesthesia, remifentanil enabled controlled hypotension, reduced middle ear blood flow and provided good surgical conditions for middle ear surgery in children [16]. In our study, although PR anesthesia produced lower surgical field visibility scores than DR anesthesia, average surgical field visibility score was < 2 under DR anesthesia, meaning no clinical difference between the two groups. According to Boezaart grading scale [12], score of 2 means slight bleeding, occasional suctioning required, and surgical field not threatened. These findings suggest that DR anesthesia is applicable for middle ear surgery and can explain why, in our otology surgical center, surgeons seldom complain of impaired surgical visibility during desflurane usage.

It is now well accepted that intraoperative blood pressure may not be correlated with bleeding at the surgical site. Hypotension alone may not improve surgical field visibility [6, 17]. By contrast, hypotension is often associated with peripheral vasodilation, which might increase bleeding and the risk of organ ischemia [12]. Therefore, we maintained mean blood pressure within ±30% change of pre-induction values in both groups. However, patients in the DR group required less remifentanil than those in the PR group, it is more likely that the reduced remifentanil requirement in the desflurane group was due to the far more potent analgesic contribution of desflurane compared with propofol, which has primarily sedative hypnotic (and minimal analgesic) effects.

Our study has some limitations. First, we did not measure blood loss, as middle ear surgery rarely results in massive blood loss. Second, subjective assessment of surgical field visibility makes comparisons to previous studies difficult. Third, we did not measure blood flow in the middle and inner ear during controlled hypotension. However, Degoute et al. [15] reports that remifentanil reduces middle ear blood flow by 25% as measured by laser Doppler flowmetry. Fourth, whether ear infection is present is a major predictor of surgical field visibility. Thus, further studies are needed to understand the effects of anesthetic agents on surgical field visibility in chronic suppurative otitis media patients undergoing middle ear surgery. Finally, due to the study population these results are limited to adults the findings in our study might require further confirmation in children.

## Conclusion

Although there is a statistically significant difference between propofol-remifentanil and desflurane-remifentanil groups with regard to surgical field visibility according to the Boezaart grading scale, when remifentanil is used in combination with desflurane, similar operative conditions are achieved. The requirement for remifentanil is greater in the desflurane-remifentanil group.

## Abbreviations

BIS: Bispectral index
HR: Heat beat
LOC: Loss of consciosness
MAP: Mean blood pressure
PACU: Post-anesthesia care unit
PONV: Postoperative nausea and vomiting
